# PD-1/PD-L1 Pathway: A Therapeutic Target in CD30+ Large Cell Lymphomas

**DOI:** 10.3390/biomedicines10071587

**Published:** 2022-07-04

**Authors:** Wei Xie, L. Jeffrey Medeiros, Shaoying Li, Guilin Tang, Guang Fan, Jie Xu

**Affiliations:** 1 Department of Pathology and Laboratory Medicine, Oregon Health & Science University, 3181 S.W. Sam Jackson Park Road, Portland, OR 97239, USA; xiew@ohsu.edu (W.X.); fang@ohsu.edu (G.F.); 2 Department of Hematopathology, The University of Texas MD Anderson Cancer Center, 1515 Holcombe Blvd, Houston, TX 77030, USA; ljmedeiros@mdanderson.org (L.J.M.); sli6@mdanderson.org (S.L.); gtang@mdanderson.org (G.T.)

**Keywords:** PD-1, PD-L1, CD30, large cell lymphoma

## Abstract

The programmed death-ligands, PD-L1 and PD-L2, reside on tumor cells and can bind with programmed death-1 protein (PD-1) on T-cells, resulting in tumor immune escape. PD-1 ligands are highly expressed in some CD30+ large cell lymphomas, including classic Hodgkin lymphoma (CHL), primary mediastinal large B-cell lymphoma (PMBL), Epstein–Barr virus (EBV)-positive diffuse large B-cell lymphoma (EBV+ DLBCL), and anaplastic large cell lymphoma (ALCL). The genetic alteration of the chromosome 9p24.1 locus, the location of *PD-L1*, *PD-L2*, and *JAK2* are the main mechanisms leading to PD-L1 and PD-L2 overexpression and are frequently observed in these CD30+ large cell lymphomas. The JAK/STAT pathway is also commonly constitutively activated in these lymphomas, further contributing to the upregulated expression of PD-L1 and PD-L2. Other mechanisms underlying the overexpression of PD-L1 and PD-L2 in some cases include EBV infection and the activation of the mitogen-activated protein kinase (MAPK) pathway. These cellular and molecular mechanisms provide a scientific rationale for PD-1/PD-L1 blockade in treating patients with relapsed/refractory (R/R) disease and, possibly, in newly diagnosed patients. Given the high efficacy of PD-1 inhibitors in patients with R/R CHL and PMBL, these agents have become a standard treatment in these patient subgroups. Preliminary studies of PD-1 inhibitors in patients with R/R EBV+ DLBCL and R/R ALCL have also shown promising results. Future directions for these patients will likely include PD-1/PD-L1 blockade in combination with other therapeutic agents, such as brentuximab or traditional chemotherapy regimens.

## 1. Introduction

**Figure 1:**
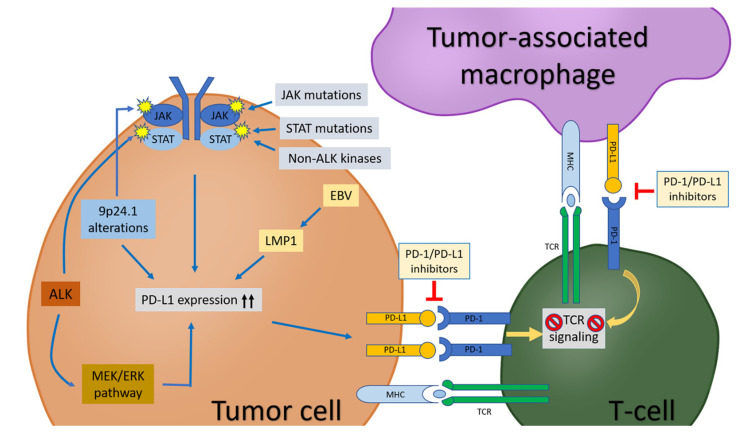
The PD-1/PD-L1 pathway in tumor cells, tumor-associated macrophages (TAMs), and T-cells in tumor microenvironment. PD-L1 expression in tumor cells is upregulated by: (1) chromosome locus 9p24.1 alterations (polysomy, copy gain, amplification, translocation, etc.); (2) activation of JAK/STAT pathway due to chromosome locus 9p24.1 alterations, kinases (such as ALK and non-ALK), *JAK/STAT* mutations; (3) EBV infection; (4) MEK/ERK pathway. PD-L1 on tumor cells and/or TAMs interact with PD-1 on T-cells, leading to inhibition of T-cell receptor (TCR) signaling pathway and subsequent T-cell “exhaustion”. Blockade of the PD-1/PD-L1 pathway can release T-cells from the inhibitory effects by tumor cells and/or TAMs and re-establish the T-cell-mediated antitumor immune response.

Programmed cell death protein (PD-1), also known as CD279, is encoded by *PDCD1* on chromosome 2q37.3 [1,2]. In 1992, PD-1 was discovered and was initially thought to be an apoptosis-associated molecule, but, subsequently, PD-1 was shown to be a key checkpoint molecule that participates in immune homeostasis by interacting with its ligands, PD-L1 (CD274 or B7-H1) and PD-L2 (CD273 or B7-DC) [3,4,5]. In cancers, PD-1 binds to PD-L1 or PD-L2 on the surface of tumor cells and/or tumor-associated macrophages (TAMs) in the tumor microenvironment, transducing inhibitory signals to the T-cell receptor (TCR) pathway [3,6]. As a result, TCR-mediated signaling activation and cellular proliferation are inhibited [7,8,9] (Figure 1). In this role, the PD-1/PD-L1 pathway plays a critical role in tumor immunity [10].

Blockade of the PD-1/PD-L1 pathway can release T-cells from the inhibitory effects of tumor cells and re-establish the-T-cell mediated antitumor immune response [11]. In recent years, great success has been achieved in the development of cancer immunotherapies, including PD1/PD-L1 blockade [6]. In 2018, Drs. James Allison (MD Anderson Cancer Center, Houston, Texas, U.S.A.) and Tasuku Honjo (Kyoto University, Kyoto, Japan) shared the Nobel Prize in Physiology or Medicine for their discoveries that enabled cancer immunotherapy [4]. Generally speaking, PD-L1 levels expressed by tumor cells are associated with a response to PD-1/PD-L1 inhibitor therapies that are widely used to treat patients with non-hematologic and hematologic malignancies, including lung cancers, melanoma, and lymphomas [12,13]. These inhibitors can prevent the binding of PD-1 to its ligands, restoring the T-cell immune response and leading to substantial and sustainable responses in patients [14]. In hematolymphoid neoplasms, the highest response rates have been achieved in patients with classic Hodgkin lymphoma (CHL) [6,15]. In contrast, variable responses have been observed in patients with non-Hodgkin lymphomas, including diffuse large B-cell lymphoma (DLBCL) and T-cell lymphomas that are known to have heterogeneous PD-L1/PD-L2 expression [6]. PD-L1 expression assessed by immunohistochemistry has been used as the main method to evaluate PD-L1 positivity on neoplastic cells. The cut-off values for PD-L1 positivity vary among studies. For example, a 5% cut-off was applied in an early study [16], whereas different cut-offs were employed in various studies of lymphomas [6,17,18]. 

CD30+ large cell lymphomas, including CHL, primary mediastinal large B-cell lymphoma (PMBL), Epstein–Barr-virus-positive (EBV+) DLBCL, and anaplastic large cell lymphoma (ALCL) show strong PD-L1 expression. In this review, we discuss the most updated studies focused on the PD-1/PD-L1 axis, as well as PD-1 blockade immunotherapy in these lymphoma types. 

## 2. Classic Hodgkin Lymphoma

CHL is characterized by a marked inflammatory infiltrate admixed with sparsely distributed (usually <10%) CD30+ large neoplastic cells, including mononuclear Hodgkin (H) cells and multinucleated Reed–Sternberg (RS) cells [19]. The inflammatory infiltrate is composed of small lymphocytes, histiocytes, eosinophils, neutrophils, and plasma cells in variable proportions and represents the tumor microenvironment [20]. HRS cells have a high-level expression of PD-L1 and PD-L2 [21]. The overexpression of PD-L1 by HRS cells has been shown by immunohistochemistry in 70–87% of CHL cases [14,22,23,24,25]. The overexpressed PD-L1 and PD-L2 on HRS cells interact with PD-1-positive cytotoxic T-cells in the microenvironment, leading to the suppression of T-cell proliferation and function [25,26]. Therefore, HRS cells take advantage of their high expression of PD-1 ligands to escape from immune surveillance [21].

An increased PD-L1 and PD-L2 expression is associated with genetic alteration on chromosome 9p24.1, where *PD-L1 (CD274)* and *PD-L2 (PDCD1LG2)* are located [21,27]. Genetic alterations such as polysomy, copy gain, and amplification on chromosome 9p24.1 play critical roles in the overexpression of PD-L1 and PD-L2 in CHL [25]. Gene fusion between the class II MHC transactivator (*CIITA*) and upstream of *PD-L1* has also been detected in 15% CHL, placing *PD-L1* under the transcriptional control of the *CIITA* promoter and driving PD-L1 overexpression [28].

In addition, the chromosome 9p24.1 amplification region in CHL often extends to include the Janus kinase 2 (*JAK2*) locus, which is located 322 kilobases upstream from *PD-L1* on 9p24.1. *JAK2* amplification resulting in an increased JAK2 expression and kinase activity leads to the activation of downstream molecules such as STAT1. The activation of JAK2/STAT1 induced PD-1 ligand transcription, further augmenting PD-1 ligand expression [27]. The JAK2/STAT signaling pathway in CHL is sensitive to JAK2 inhibitors [29,30,31]. Furthermore, approximately 90% of CHL cases exhibit JAK/STAT pathway dysregulation caused by alterations in multiple genes, including *JAK1*, *JAK2*, *STAT3*, and *STAT5B*, supporting a pivotal role of this pathway in CHL [32].

A subset of CHL cases are positive for EBV infection [33]. Strikingly, 40 of 41 EBV+ CHL cases showed an increased PD-L1 expression in one study [34]. EBV infection is another, not fully understood, mechanism underlying PD-L1 overexpression [35]. EBV induces PD-L1 overexpression by activating the activator protein-1 (AP-1) and JAK/STAT signaling pathways [35]. In EBV+ CHL with a normal 9p24.1 copy number, the constitutive activation of the AP-1 pathway has been detected; in these cases, AP-1 is bound to an AP-1-responsive enhancer within *PD-L1*, resulting in increased *PD-L1* promoter activity [35]. 

In addition to HRS cells, PD-L1/L2 is also expressed on TAMs in CHL. TAMs suppress cytotoxic T-cells and exert tumor-promoting and immunosuppressive functions [21,36]. A high number of TAMs in CHL independently predict inferior failure-free and overall survival (OS) in CHL patients [37]. Increased TAMs are also associated with primary treatment failure, shortened progression-free survival (PFS), and an increased likelihood of disease relapse after autologous stem cell transplantation (ASCT) [36]. Immune evasion via PD-1/PD-L1 on monocyte/macrophages has been shown to be prominent in CHL [38]. PD-L1/L2 expression was elevated in monocytes co-cultured with HRS cells within one hour, but not in monocytes cultured with supernatants of HRS cells [39]. An immunofluorescence analysis of PD-L1/L2 revealed that their upregulation results in membrane transfer called “trogocytosis” from HRS cells to monocytes. In CHL patients, PD-L1 and PD-L2 are upregulated in TAMs in contact with HRS cells, but not in TAMs distant from HRS cells, suggesting that trogocytosis occurs in CHL. These findings further suggest that trogocytosis may be a mechanism that induces the rapid upregulation of PD-L1/L2 by monocytes in CHL to evade antitumor immunity. The upregulated expression of PD-L1 in TAMs is also induced by interferon-gamma [40]. PD-L1+ TAMs can interact with PD-1+ CD4+ T-cells, leading to the dysfunction of CD4+ T-cells via PD-L1/PD-1 interaction and/or preventing their direct access to HRS cells [40]. 

The standard therapy for newly diagnosed CHL patients in the U.S.A. has been the doxorubicin, bleomycin, vinblastine, and dacarbazine (ABVD) chemotherapy regimen, although recent studies have replaced bleomycin with brentuximab vedotin (BV), an anti-CD30 antibody linked to a toxin [41,42]. Using ABVD, relapse occurs in a subset of patients, including up to one third of CHL patients with advanced-stage disease [43]. In patients with relapsed/refractory (R/R) CHL, the standard treatment has been high-dose chemotherapy followed by ASCT [44]. In ASCT-ineligible CHL patients, BV has been shown to have activity and is approved by the U.S. Food and Drug Administration (FDA). Not all patients can tolerate BV or respond sufficiently to BV, and, therefore, novel therapies are needed for this subset of CHL patients. 

Based on strong PD-L1 expression by HRS cells and TAMs in CHL, CHL patients were treated with PD-1/PD-L1 inhibitors, and this approach has been proven to be highly successful. The PD-1 inhibitors, nivolumab and pembrolizumab, were approved by the United States Food and Drug Administration (FDA) in 2016 and 2017, respectively, as therapies for transplant-ineligible R/R CHL patients [21]. Nivolumab was the first PD-1 inhibitor used in patients with R/R CHL [45]. In a phase I study, nivolumab was highly effective in heavily treated R/R CHL patients, with an overall response rate (ORR) of 87% and a PFS rate of 86% at 24 weeks [45]. In another multicenter, multicohort, single-arm phase II trial study, nivolumab resulted in frequent responses with an acceptable safety profile in CHL patients who progressed after ASCT and BV [22]. Pembrolizumab, another PD-1 inhibitor, was reported to induce good responses in R/R CHL patients in a phase II, multicenter, single-arm study [46,47]. In this study, 210 heavily pretreated patients were treated with pembrolizumab, with an ORR of 71.9%, a complete response rate (CRR) of 27.6%, and a median duration of response of 16.5 months [46,47]. 

In a multi-center, phase III study of patients aged 18 years or older with R/R CHL who were ineligible for ASCT or who had relapsed after ASCT, 151 patients were randomly assigned to pembrolizumab and 153 to BV [48]. After a median follow-up time of 25.7 months, the median PFS was 13.2 months for the pembrolizumab group, significantly higher than the 8.3 months in the BV group. These data suggest pembrolizumab as a preferred treatment option for patients with R/R CHL who have relapsed after ASCT or who are ineligible for ASCT. 

Other PD-1 inhibitors, such as intilimab, camrelizumab, and tislelizumab, and PD-L1 inhibitors such as avelumab may also be used in R/R CHL patients [49,50]. In a phase II study of camrelizumab, 76% of R/R CHL patients had an objective response, including 28% who underwent a complete remission [51]. In the JAVELIN Hodgkins study, avelumab was used to treat R/R CHL patients. The ORR in all randomized patients was 49.1%, with a CRR of 19.4% [52]. These findings indicate that these PD-1/PD-L1 inhibitors have strong antitumor activity in R/R CHL patients and that some patients have a durable response. 

PD-1 blockade has also been investigated in combination with chemotherapy for the treatment of newly diagnosed or R/R CHL. In patients ≥ 18 years of age with newly diagnosed, untreated, early, unfavorable, or advanced-stage disease, brief pembrolizumab monotherapy followed by AVD was highly effective and safe [53]. In a phase I/II study in which nivolumab was administered in combination with BV as an initial salvage treatment in patients with R/R CHL, the ORR was 82% and the CRR was 61% [54]. These findings suggest that the combination of PD-1/PD-L1 inhibitors with other therapies is a potential alternative to traditional chemotherapy. 

The high response rates of PD-1 blocking antibodies in R/R CHL patients confirm the critical role of PD-1/PD-1 ligand interactions in this disease. The data from a recent study indicate that blocking PD-L1 reverse signaling may be another mechanism of high response with PD-1/PD-L1 inhibitors in CHL patients. PD-L1 on CHL cell lines could be stimulated by an agonistic monoclonal antibody, resulting in increased cell growth and decreased apoptosis [5]. Interestingly, soluble PD-1 levels in the serum of CHL patients were significantly elevated compared with healthy people. PD-1, both membrane-bound and soluble forms, induced PD-L1 reverse signaling in CHL cell lines, which could be inhibited by nivolumab [5]. 

In summary, frequent genetic alterations of the chromosome 9p24.1 locus (amplification, copy gain, polysomy, rearrangement), constitutive activation of the JAK/STAT pathway, and EBV infection lead to PD-L1/L2 overexpression in CHL and resultant tumor evasion from immune surveillance (Table 1). These mechanisms provide a scientific rationale for treating R/R CHL patients with PD-1/PD-L1 inhibitors, which has become a standard of care for this patient population [15]. Furthermore, synergistic effects of PD-1/PD-L1 inhibitors and chemotherapy or BV have been observed in newly diagnosed or R/R CHL patients, respectively, indicating that PD-1/PD-L1 blockade in combination with other therapeutic agents may be a future strategy. 

## 3. Diffuse Large B-Cell Lymphoma (DLBCL)

DLBCL is a biologically and clinically heterogeneous group of neoplasms [19]. In 2000, gene expression profiling analysis was used to subdivide cases of DLBCL into germinal center B-cell-like (GCB), activated B-cell (ABC), and unclassified subtypes [20,55]. As gene expression profiling is not widely available, others subsequently used surrogate immunohistochemistry algorithms to subdivide DLBCL cases into GCB and non-GCB groups. Although this system contributed to the understanding of DLBCL, these approaches were too simplistic to capture the true heterogeneity of DLBCL. More recent studies from the National Institutes of Health, Harvard, and the United Kingdom have divided DLBCL cases into up to six groups [56,57]. Although these studies differ in methods and somewhat in their results, there is also similarity between these studies, indicating that consensus on various molecular subsets of DLBCL is emerging. To date, the expression of PD-L1 has been shown in 11–31% of DLBCL cases [16,17,18,24,58,59,60], being more frequent in the ABC type (up to 45%) than in the GCB type (15%) [61]. PD-L1 expression is also more commonly detected in EBV+ DLBCL compared with EBV-negative cases [6].

Similar to CHL, an increased PD-L1 expression in a subset of DLBCL cases is associated with alterations of chromosome 9p24.1, such as copy number gains, amplification, and translocation, but these alterations are present at a much lower frequency than in CHL [14,61]. Structural variations (SVs) that commonly disrupt the 3’ region of *PD-L1* were found in 8% of DLBCL cases in one study [62]. Kataoka et al. found that these SVs consistently led to elevated aberrant *PD-L1* transcripts that were stabilized by truncation of the 3’-untranslated region (UTR). The disruption of the *PD-L1* 3’-UTR in an animal model facilitated the immune evasion of EG7-OVA tumor cells, with an increased PD-L1 expression in vivo, which was effectively blocked by a PD-1/PD-L1 inhibitor [62].

Gene alterations and PD-L1 protein expression have been closely linked with phosphorylated STAT3 (pSTAT3) expression in DLBCL [63]. Phosphorylated STAT3 has been observed in 16% of DLBCL and was associated with the non-GCB/ABC subtype [64,65], suggesting that the JAK/STAT pathway may play a role in upregulating PD-L1 expression in DLBCL. Elevated serum IL-10 levels seen in some DLBCL patients can also induce JAK/STAT activation [66]. 

The standard treatment of DLBCL patients is currently rituximab, cyclophosphamide, doxorubicin, vincristine, and prednisolone (R-CHOP) [67,68]. However, new therapeutic approaches are needed for R/R DLBCL patients who generally have a poor prognosis. Unlike the situation in CHL patients, the efficacy of PD-1 blockade in trials of DLBCL patients has been disappointing. For example, among 121 patients with R/R DLBCL who were ineligible for ASCT and received nivolumab, the response rate was only 10%, and the median duration was less than 1 year [69]. The authors concluded that nivolumab monotherapy is associated with a low ORR among patients with DLBCL who are ineligible for or had failed ASCT [69]. The low response rate of PD-1/PD-L1 inhibitors in DLBCL patients may be explained by the heterogeneity in the prevalence of PD-L1 expression [7]. Although the monotherapy of PD-1/PD-L1 inhibitors is not adequate, the potential combination of PD-1/PD-L1 inhibitors with other therapies may be an effective approach to treat R/R DLBCL patients [70,71]. 

### 3.1. Primary Mediastinal Large B-Cell Lymphoma

Although PD-L1 expression is generally low in unselected cases of DLBCL, PD-L1 expression is high in some subtypes of DLBCL, such as PMBL, which accounts for approximately 10% of DLBCL cases [72]. There are overlapping morphological and immunophenotypic features between PMBL and CHL. Gene expression profiling has shown that PMBL is closely related to CHL [73,74]. The biologic hallmarks of PMBL are reminiscent of CHL, including the activation of the JAK/STAT and NF-kB signaling pathways, as well as an immune evasion phenotype through multiple converging genetic aberrations [75]. Similar to CHL, PMBL shows an overexpression of PD-L1/L2 and usually CD30, but PMBL has a stronger PD-L2 expression and variable CD30 expression compared with CHL. 

PD-L1 expression in PMBL as assessed by immunohistochemistry is high, ranging from 36% to 100% of cases [14,16,24,59]. Similar to CHL, amplification and copy number gain are the major mechanisms that upregulate the expression of PD-L1 and PD-L2 in PMBL. An amplification of the 9p24.1 locus is detected in up to 70% of PMBL cases [27,76]. Copy number gains of chromosome 9p24.1 are observed in 29–55% of PMBL cases [27,76]. In addition, translocations involving 9p24.1 are found in 20% of PMBL cases [77,78]. A relatively high frequency of 9p24.1 translocation is a unique feature of PMBL. Gene fusions involving *CIITA* and upstream of *PD-L1* are also commonly detected in PBML (38%) [28]. PMBL with 9p24.1/*JAK2* copy gain is sensitive to JAK2 inhibition in in vitro and in vivo, supporting the activation of the JAK/STAT pathway in PMBL [29]. The JAK/STAT pathway in PMBL is also activated by several other mechanisms, including activating mutations of *STAT6* and *IL-4R*, inactivating mutations of negative regulators of the JAK/STAT pathway (such as *SOCS-1* and *PTPN1*), and paracrine activation by IL-13 [79].

Refractory disease can occur in up to 10% of patients with PMBL and correlates with poor outcomes. Emerging data support the high efficacy of PD-1 inhibitors in PMBL patients. Two studies reported good response rates in R/R PMBL patients treated with pembrolizumab, with an ORR of 41% and 45%, respectively [80,81]. Soon after these reports, pembrolizumab was approved by the FDA for treating patients with R/R PMBL. Studies to investigate the possible role of PD-1 inhibitors as a frontline therapy in PMBL patients are currently underway.

Despite the expression of CD30 in ~80% of PMBL cases, BV has been ineffective as a single agent in patients with this disease [82]. However, nivolumab combined with BV demonstrated high antitumor activity in patients with R/R PMBL. In a phase I/II study of 30 patients with R/R PMBL who were previously treated with either ASCT or ≥ two prior chemotherapy regimens, if ineligible for ASCT, nivolumab combined with BV resulted in an ORR of 73% with a 37% CRR at a median follow-up of 11.1 months [83]. Therefore, the combination of nivolumab plus BV may represent a promising option for patients with R/R PMBL. 

### 3.2. EBV+ Diffuse Large B-Cell Lymphoma, NOS

EBV+ DLBCL not otherwise specified (NOS) accounts for <5%–15% of DLBCL cases [19]. EBV+ DLBCL is most common in elderly patients (≥50 years of age) but can occur in younger patients. EBV+ DLBCL of the elderly and in young patients may be considered as two different pathogenic types: the former being related to physiological immunosenescence and the latter being related to immune escape [84]. Elderly patients with EBV+ DLBCL NOS usually have a gradual deterioration in their immune functions due to aging, which accelerates the imbalance between the inflammatory and anti-inflammatory process. As a result, this imbalance leads to a chronic pro-inflammatory status, which can facilitate lymphomagenesis [84]. In contrast, immune escape is observed in younger patients with EBV+ DLBCL NOS. The mechanisms involved in the neoplasms include the recruitment of regulatory T-cells and production of immunosuppressive cytokines, as well as the expression of immune checkpoint proteins, such as PD-L1. Elderly patients with EBV+ DLBCL have a significantly shorter OS than younger patients [85]. Studies have shown that the genomic features of EBV+ DLBCL cases are distinct from EBV-negative DLBCL cases. There are relatively fewer genomic alterations in EBV+ DLBCL compared with EBV-negative DLBCL [86]. Others have reported that the host immune response is a crucial molecular signature and that genes associated with the B-cell receptor signaling pathway are downregulated in EBV+ DLBCL [86]. EBV+ DLBCL cases also exhibit a genetic profile different from EBV-negative DLBCL, characterized by frequent mutations in *TET2* and *DNMT3A* and infrequent mutations in *CD79B*, *MYD88*, *CDKN2A*, and *FAS* [87].

EBV+ DLBCL and CHL show overlapping histologic features [19]. Morphologically, these neoplasms are composed of large transformed cells, namely immunoblast-like cells and/or Hodgkin/Reed–Sternberg-like cells [19]. EBV+ DLBCL cases are frequently positive for CD30, much more often than EBV-negative cases [88,89]. One study reported CD30 positivity in almost all EBV+ DLBCL cases (98%) assessed [90]. In another study, CD30 expression was found in 43% of EBV+ DLBCL cases, but only in 16% of EBV-negative DLBCL cases [89]. Given the high expression of CD30, BV may be a potential treatment for EBV+DLBCL patients. In a small phase II study, the clinical activity of BV in R/R EBV+ and CD30+ non-Hodgkin lymphomas (22 mature NK/T cell and 3 mature B-cell lymphoma cases) was substantial and durable, with an ORR of 48% and a duration of response of 10.1 months, suggesting BV as a promising therapy for patients with EBV+ DLBCL [91].

EBV+ DLBCL cases often show higher levels of PD-L1 expression (77–100%) compared with EBV-negative cases [16,63,85]. The expression of PD-L1 in both the neoplastic cells and the microenvironment (mPD-L1) in DLBCL is significantly associated with EBV positivity. PD-L1 and mPD-L1 expression were noted in 16% and 41%, respectively, of patients with EBV+ DLBCL [17,92]. *PD-L1/PD-L2* aberrations were detected in 19% of EBV+ DLBCL cases [87]. The chromosome 9p24.1 locus was one of the most frequent sites of copy number alterations (>30%) in EBV+ DLBCL, and PD-L2 is a key target of the gains detected at the chromosome 9p24.1 locus. Chromosomal gains at 9p24.1 have been associated with a poorer OS in EBV+ DLBCL patients, suggesting that the upregulation of *PD-L2* promotes immune evasion [86]. 

At least three cellular signaling pathways are upregulated in EBV+ DLBCL: AP-1, JAK/STAT, and NF-kB [16,89,93,94]. The EBV infection of DLBCL cell lines induces a high activation of the JAK/STAT and NF-κB pathways [94]. An enhanced activation of the AP-1 and JAK/STAT pathways also likely contributes to the overexpression of PD-L1. EBV+ HIV-associated DLBCL has been reported to be enriched for *STAT3* mutations [95]. EBV+ DLBCL also showed a significantly lower expression of CIITA, MHC II, and B-cell receptor (BCR), but an overexpression of PD-L1, compared with EBV-negative DLBCL. Genetic aberrations involving *CIITA* were also more common in EBV+ DLBCL, with 23% break-apart and 6% deletion events, compared with 2% break-apart and 0% deletion events in EBV-negative DLBCL [96]. These findings suggest that antigen capture and presentation are disrupted, and that T-cell inhibitory molecules are hijacked in EBV+ DLBCL, possibly contributing to the immune escape of this disease.

PD-1 blockade has been shown to restore functions of T-cells in EBV+ DLBCL in vitro [97]. Targeting the PD-1/PD-L1 pathway may represent a potential therapeutic approach for EBV+DLBCL patients. A patient with refractory EBV+ DLBCL associated with secondary hemophagocytic syndrome has been successfully treated by a sequential combination regimen of PD-1 blockade and chimeric antigen receptor T-cells [98]. In a phase II study of nivolumab in R/R EBV+ DLBCL patients, preliminary data showed an ORR of 50% (1/2) and a CRR of 50% (1/2) [99].

In summary, genetic alterations and the expression of PD-L1 are low in non-selected cases of DLBCL but are frequent in some CD30+ types, such as PMBL and EBV+ DLBCL (Table 1). Both genetic aberrations of chromosome locus 9p24.1 and activation of the JAK/STAT pathway upregulate the expression of PD-L1 in PMBL and EBV+ DLBCL. Currently available data on blocking PD-1/PD-L1 in non-selected DLBCL patients have been disappointing so far, but PD-1 inhibitors have been very successful in treating patients with R/R PMBL, leading to their rapid approval by the FDA. Studies that investigate the possible role of PD-1 inhibitors as an up-front treatment in PMBL patients are underway. Preliminary data also suggest that PD-1 blockade is a promising therapy for EBV+ DLBCL patients.

## 4. Anaplastic Large Cell Lymphoma

ALCL is a systemic CD30+ peripheral T-cell lymphoma characterized by large pleomorphic lymphoma cells with horseshoe-shaped nuclei (so-called hallmark cells) [19]. ALCL is further classified into ALK+ and ALK-negative types. The most common translocation in ALK+ ALCL, i.e., t(2;5)(p23;q35), was discovered in the 1980s and, in 1994, Morris and Look identified the involved genes, *ALK* and *NPM1*, which form an *NPM1*::*ALK* fusion [100,101,102]. This fusion leads to an increased ALK expression and activation of downstream signaling pathways, such as JAK/STAT [103]. ALK-negative ALCL is more heterogeneous, with 20–30% of cases associated with *DUSP22* rearrangement and approximately 5–8% of cases associated with *TP63* rearrangement [104,105].

The expression of PD-L1 in ALCL ranges from 50% to 80% [59,106,107], with a higher positivity rate in ALK+ ALCL [59,106,107,108]. PD-L1 expression is associated with ALK positivity in ALCL patient specimens [107,109]. Using a 5% cut-off for PD-L1 positivity, PD-L1 expression was found in 76% of ALK+ ALCL cases versus 42% of ALK-negative ALCL cases in one large study [107]. Among ALK-negative ALCL, *DUSP22*-rearranged ALK-negative ALCL cases usually lack pSTAT3 and PD-L1 expression [110,111,112]. In addition, these cases also show a high expression of the costimulatory molecules CD58 and HLA class II, suggesting that ALK-negative ALCL may be more immunogenic when associated with *DUSP22* rearrangement [112]. 

An earlier study showed that the most significant gain in ALCL was at the chromosome 9p24.1 locus [113], but a later study reported no *PD-L1* amplification in ALK+ or ALK-negative ALCL by FISH analysis [114]. FISH studies that assessed *PD-L1/2* were performed in 25 ALCL cases: 5 cases showed polyploidy (three to four copies); but there was no evidence of rearrangements, deletions, gains, nor amplification of the *PD-L1* locus, suggesting that PD-L1 expression in ALCL may not be related to *PD-L1* amplification or rearrangement [108].

At least two signaling pathways are involved in regulating PD-L1 expression in ALCL. First, JAK/STAT3 signaling is the central pathway in ALCL pathogenesis [107]. The constitutive activation of this pathway has been observed in both ALK+ and ALK-negative ALCL [107]. Nuclear pSTAT3 expression is elevated in approximately 85% of ALK+ ALCL cases and in half of ALK-negative ALCL cases [115]. The overexpression of ALK activates the downstream transcription factor STAT3 and thus increases PD-L1 expression in ALK+ALCL tumor cells [106]. Activating mutations of *JAK1* and/or *STAT3* have been reported in approximately 20% of ALK-negative ALCL [103]. Rearrangements of other STAT3-activating kinases, such as *ROS1* or *TYK2*, were also identified in approximately 20% of ALK-negative ALCL. *STAT3* mutations have been associated with STAT3 activation in ALK-negative ALCL [116]. Another possible pathway regulating PD-L1 expression in ALCL is the MEK/ERK signaling pathway [117]. The activation of the MEK/ERK pathway can promote ALCL cell proliferation and survival [118]. *NPM1*::*ALK* fusion has been shown to activate the STAT3 pathway and signalosome (GRB2/SOS1), resulting in the activation of the MEK/ERK pathway and inducing PD-L1 expression [12]. 

Limited studies have been performed to investigate the efficacy and safety of PD-1/PD-L1 blockade in ALCL patients. However, a few case reports have shown the effectiveness of PD-1 blockade in patients with R/R ALCL [14]. In these case reports, all patients with R/R ALCL disease responded well to PD-L1 inhibitors [119,120,121]. In the report by Chan and colleagues, a patient with stage IV ALK-negative ALCL and unknown PD-L1 expression status relapsed after being treated with multiple lines of therapy, including chemotherapy, BV, and SCT. The patient was treated with pembrolizumab and achieved complete remission. The patient remained asymptomatic 18 weeks after the initiation of pembrolizumab therapy [119]. In a report by Rigaud and colleagues, a patient with relapsed ALK+ ALCL who was strongly positive for PD-L1 positivity did not respond to chemotherapy or ALK inhibitors. After starting on nivolumab, the patient had a dramatic clinical improvement and remained in complete remission for at least 18 months [120]. Hebart et al. reported a patient with R/R ALK+ ALCL (PD-L1+) who received multiple therapies (chemotherapy, BV, ALK inhibitor, and SCT). The patient relapsed with macrophage activation syndrome and did not respond to an ALK inhibitor or BV. After starting on nivolumab, the patient rapidly improved, with the disappearance of the macrophage activation syndrome. The patient was negative for the disease 8 months after the start of nivolumab [121]. Further, preliminary data of a phase II study of pembrolizumab in combination with romidepsin in patients with R/R T-cell lymphomas, including ALCL, showed an ORR of 50% that was durable and had an acceptable safety profile. Patients achieving complete remission included those patients whose neoplasms had higher levels of PD-L1 expression. Moreover, these patients also maintained a longer duration of response compared with prior therapies. This study was presented at the American Society of Hematology 2020 meeting [122]. A clinical trial of nivolumab for pediatric and adult relapsing/refractory ALK+ ALCL patients is currently being performed, and results are not yet available (NCT03703050). 

PD-1/PD-L1 blockade can overcome PD-1/PD-L1-mediated T-cell anergy and promote the proliferation of anti-tumor T-cells and restore T-cell immunity. On the other hand, PD-1/PD-L1 blockade may also activate neoplastic T-cells when treating T-cell neoplasms. Two patients with R/R T-cell lymphomas experienced hyperprogression within the first 10 days of treatment with pembrolizumab and romidepsin [122]. Hyperprogression is a rare syndrome characterized by a rapidly progressing tumor volume and early fatality, quite different from pseudo-progression, which is a short-term increase in tumor size attributable to a successful anti-tumor immune response (NCT02631746) [123]. Nivolumab has led to the rapid progression of disease in patients with adult T-cell leukemia/lymphoma (31467059). In addition, peripheral T-cell lymphoma, NOS, has been reported in a patient with a metastatic epithelial neoplasm after being treated with pembrolizumab [124]. According to the FDA Adverse Event Reporting System (FAERS) from 2012–2018, 12 cases of T-cell lymphoma have been reported in patients after PD-1 inhibitor treatment [109,124]. The frequency of T-cell lymphoma secondary to immune checkpoint inhibitors (pembrolizumab, nivolumab, and ipilimumab) was 0.02%, with a 17% mortality rate; the probability of the risk of T-cell lymphoma compared with other drugs in a pharmacovigilance database was increased at 1.91 [124]. A long-term follow-up of patients who receive checkpoint inhibitors and further investigation into T-cell lymphoma risk are needed. 

### Breast Implant-Associated ALCL

Breast-implant-associated ALCL (BI-ALCL) is a rare type of ALCL arising around textured-surface breast implants. Although BI-ALCL is morphologically and immunophenotypically indistinguishable from systemic ALCL [19], BI-ALCL does not have genomic alterations typically seen in systemic ALCL, such as rearrangements of *ALK*, *DUSP22*, and *TP63* [125]. Outcomes of the patients with BI-ALCL are generally favorable, with most patients presenting with an isolated periprosthetic effusion. However, a small subset of patients developed disseminated disease [126,127]. The JAK/STAT pathway is important in the pathogenesis of BI-ALCL. All BI-ALCL cases tested have been positive for pSTAT3 [125,128], supporting the activation of the JAK/STAT signaling pathway. The constitutive activation of the JAK/STAT3 pathway in some BI-ALCL cases is associated with recurrent somatic mutations of *JAK1* and/or *STAT3*. Sequence variants leading to JAK/STAT activation were identified in 10 of 11 BI-ALCL cases in one study [129]. In another study, 59% of BI-ALCL cases showed mutations in ≥ one member of the JAK/STAT pathway, including *STAT3* (38%), *JAK1* (18%), and *STAT5B* (3%), and in negative regulators of this pathway, such as *SOCS3* (6%), *SOCS1* (3%), and *PTPN1* (3%) [128]. A *STAT3*-*JAK2* fusion has also been identified in a case of BI-ALCL [130]. A recent study showed PD-L1 expression and *PD-L1* copy number alterations in 56% and 33% of BI-ALCL cases, respectively [131]. The activation of the JAK/STAT pathway in BI-ALCL likely contributes to PD-L1 expression. Given this high expression, PD-1 blockade is a potential therapeutic strategy for these patients. At the time of writing, there are no published studies in which PD-1 blockade was evaluated in BI-ALCL patients.

In summary, the expression of PD-L1 in ALCL is associated with ALK positivity and JAK/STAT3 activation (Table 1). In contrast to the other CD30+ large B-cell lymphomas, genomic aberrations of the 9p24.1 chromosome locus are rare in ALCL. Therefore, PD-L1 expression is mainly upregulated by constitutive action of the JAK/STAT pathway. Preliminary studies have shown the promising efficacy of PD-1 inhibitors in R/R ALCL patients. At the same time, there is a concern that PD-1 pathway blockade could accelerate the growth of T-cell lymphomas in rare cases. The benefits and safety of PD-1 inhibitors in ALCL patients require further investigation.

## 5. Conclusions

Large cell lymphomas are traditionally treated with intensive chemotherapy, with or without ASCT. The past decades have witnessed significant advances in tumor immunotherapy; in particular, in the recognition and understanding of PD-1/PD-L1 axis blockade. The efficacy of PD-1/PD-L1 inhibitors is highly associated with the expression levels of PD-L1 and PD-L2 by tumor cells. Understanding the mechanisms of the PD-1/PD-L1 pathway is critical for the appropriate and selective application of PD-1 inhibitors in patients who may respond well. CHL, PMBL, EBV+ DLBCL, and ALCL share common features, such as CD30 positivity, PD-L1 and/or PD-L2 expression, chromosome 9p24.1 alterations, JAK/STAT activation, and EBV infection (Table 1; Figure 1). Therefore, patients with these neoplasms may need to be evaluated for PD-L1 expression, as they may be good candidates for PD-1/PD-L1 inhibitors, alone or in combination with BV or other therapies. PD-1 inhibitors have also proven to be very successful in treating R/R CHL and R/R PMBL patients. Additional studies are currently underway or will be needed to investigate the role of these inhibitors in the treatment of patients with other types of R/R lymphomas, as well as newly diagnosed patients. 

## Figures and Tables

**Table 1:** PD-1/PD-L1 pathway and its blockade in CD30+ large cell lymphomas.

CD30+ Large Cell Lymphoma	CHL	PMBL	EBV+ DLBCL	Systemic ALCL/ BI-ALCL
**PD-L1/PD-L2 Protein Expression on Tumor Cells**	High	High, particularly PD-L2	High	Higher in ALK+ than ALK-cases; Negative in *DUSP22*-R cases
**9p24.1 Alterations (Amplification, Copy Gain, Polysomy, Translocation)**	Yes	Yes (mainly PD-L2)	Yes (mainly PD-L2)	Rare
**Activation of JAK/STAT Pathway**	Yes	Yes	Yes	Yes
**JAK/STAT Mutation**	Yes	Yes	Yes	Yes
**JAK Rearrangement**				Yes (BI-ALCL)
**Non-JAK Tyrosine Kinase Alteration**				*ROS1*-R, *TYK*-R
**EBV-Induced AP-1 Activation**	Yes (EBV+ cases)		Yes	
**Activation of MEK/ERK Pathway**				Yes
**PD-1/PD-L1 Blockade Therapy**				
**Single Agent**	Approved for R/R disease	Approved for R/R disease; Investigation going on in up-front setting	Promising in R/R disease (preliminary studies)	Promising in R/R systemic ALCL disease (preliminary studies)
**Combined with BV**	Effective in R/R disease	Effective in R/R disease		
**Combined with Chemotherapy**	Effective in newly diagnosed patients			

ALCL, anaplastic large cell lymphoma; BI-ALCL, breast-implant-associated ALCL; BV, brentuximab vedotin; CHL, classic Hodgkin lymphoma; DLBCL, diffuse large B-cell lymphoma; PMBL, primary mediastinal large B-cell lymphoma; R, rearrangement; R/R, relapsed/refractory; EBV, Epstein–Barr virus.

## Data Availability

Not applicable.

## References

[1] Zak K.M., Grudnik P., Magiera K., Domling A., Dubin G., Holak T.A. (2017). Structural Biology of the Immune Checkpoint Receptor PD-1 and Its Ligands PD-L1/PD-L2. Structure.

[2] Alsaab H.O., Sau S., Alzhrani R., Tatiparti K., Bhise K., Kashaw S.K., Iyer A.K. (2017). PD-1 and PD-L1 Checkpoint Signaling Inhibition for Cancer Immunotherapy: Mechanism, Combinations, and Clinical Outcome. Front. Pharmacol..

[3] Ishida Y., Agata Y., Shibahara K., Honjo T. (1992). Induced expression of PD-1, a novel member of the immunoglobulin gene superfamily, upon programmed cell death. EMBO J..

[4] Ishida Y. (2020). PD-1: Its Discovery, Involvement in Cancer Immunotherapy, and Beyond. Cells.

[5] Jalali S., Price-Troska T., Bothun C., Villasboas J., Kim H.J., Yang Z.Z., Novak A.J., Dong H., Ansell S.M. (2019). Reverse signaling via PD-L1 supports malignant cell growth and survival in classical Hodgkin lymphoma. Blood Cancer J..

[6] Xu-Monette Z.Y., Zhou J., Young K.H. (2018). PD-1 expression and clinical PD-1 blockade in B-cell lymphomas. Blood.

[7] Garcia-Lacarte M., Grijalba S.C., Melchor J., Arnaiz-Leche A., Roa S. (2021). The PD-1/PD-L1 Checkpoint in Normal Germinal Centers and Diffuse Large B-Cell Lymphomas. Cancers.

[8] Latchman Y., Wood C.R., Chernova T., Chaudhary D., Borde M., Chernova I., Iwai Y., Long A.J., Brown J.A., Nunes R. (2001). PD-L2 is a second ligand for PD-1 and inhibits T cell activation. Nat. Immunol..

[9] Xia Y., Medeiros L.J., Young K.H. (2016). Immune checkpoint blockade: Releasing the brake towards hematological malignancies. Blood Rev..

[10] Bardhan K., Anagnostou T., Boussiotis V.A. (2016). The PD1:PD-L1/2 Pathway from Discovery to Clinical Implementation. Front. Immunol..

[11] Azuma T., Yao S., Zhu G., Flies A.S., Flies S.J., Chen L. (2008). B7-H1 is a ubiquitous antiapoptotic receptor on cancer cells. Blood.

[12] Zhang J.P., Song Z., Wang H.B., Lang L., Yang Y.Z., Xiao W., Webster D.E., Wei W., Barta S.K., Kadin M.E. (2019). A novel model of controlling PD-L1 expression in ALK(+) anaplastic large cell lymphoma revealed by CRISPR screening. Blood.

[13] Herbst R.S., Soria J.C., Kowanetz M., Fine G.D., Hamid O., Gordon M.S., Sosman J.A., McDermott D.F., Powderly J.D., Gettinger S.N. (2014). Predictive correlates of response to the anti-PD-L1 antibody MPDL3280A in cancer patients. Nature.

[14] Xie W., Medeiros L.J., Li S., Yin C.C., Khoury J.D., Xu J. (2020). PD-1/PD-L1 Pathway and Its Blockade in Patients with Classic Hodgkin Lymphoma and Non-Hodgkin Large-Cell Lymphomas. Curr. Hematol. Malig. Rep..

[15] Ansell S.M. (2021). PD-1 Blockade in Classic Hodgkin Lymphoma. JCO Oncol. Pract..

[16] Chen B.J., Chapuy B., Ouyang J., Sun H.H., Roemer M.G., Xu M.L., Yu H., Fletcher C.D., Freeman G.J., Shipp M.A. (2013). PD-L1 expression is characteristic of a subset of aggressive B-cell lymphomas and virus-associated malignancies. Clin. Cancer Res..

[17] Kiyasu J., Miyoshi H., Hirata A., Arakawa F., Ichikawa A., Niino D., Sugita Y., Yufu Y., Choi I., Abe Y. (2015). Expression of programmed cell death ligand 1 is associated with poor overall survival in patients with diffuse large B-cell lymphoma. Blood.

[18] Xing W., Dresser K., Zhang R., Evens A.M., Yu H., Woda B.A., Chen B.J. (2016). PD-L1 expression in EBV-negative diffuse large B-cell lymphoma: Clinicopathologic features and prognostic implications. Oncotarget.

[19] Swerdlow S.H., Campo E., Harris N.L., Jaffe E.S., Pileri S.A., Stein H., Thiele J. (2017). WHO Classification of Tumours of Haematopoietic and Lymphoid Tissues.

[20] Muenst S., Hoeller S., Dirnhofer S., Tzankov A. (2009). Increased programmed death-1+ tumor-infiltrating lymphocytes in classical Hodgkin lymphoma substantiate reduced overall survival. Hum. Pathol..

[21] Al-Hadidi S.A., Lee H.J. (2021). Checkpoint Inhibition Therapy in Transplant-Ineligible Relapsed or Refractory Classic Hodgkin Lymphoma. JCO Oncol. Pract..

[22] Younes A., Santoro A., Shipp M., Zinzani P.L., Timmerman J.M., Ansell S., Armand P., Fanale M., Ratanatharathorn V., Kuruvilla J. (2016). Nivolumab for classical Hodgkin's lymphoma after failure of both autologous stem-cell transplantation and brentuximab vedotin: A multicentre, multicohort, single-arm phase 2 trial. Lancet Oncol..

[23] Jelinek T., Mihalyova J., Kascak M., Duras J., Hajek R. (2017). PD-1/PD-L1 inhibitors in haematological malignancies: Update 2017. Immunology.

[24] Menter T., Bodmer-Haecki A., Dirnhofer S., Tzankov A. (2016). Evaluation of the diagnostic and prognostic value of PDL1 expression in Hodgkin and B-cell lymphomas. Hum. Pathol..

[25] Roemer M.G., Advani R.H., Ligon A.H., Natkunam Y., Redd R.A., Homer H., Connelly C.F., Sun H.H., Daadi S.E., Freeman G.J. (2016). PD-L1 and PD-L2 Genetic Alterations Define Classical Hodgkin Lymphoma and Predict Outcome. J. Clin. Oncol..

[26] Mottok A., Steidl C. (2018). Biology of classical Hodgkin lymphoma: Implications for prognosis and novel therapies. Blood.

[27] Green M.R., Monti S., Rodig S.J., Juszczynski P., Currie T., O'Donnell E., Chapuy B., Takeyama K., Neuberg D., Golub T.R. (2010). Integrative analysis reveals selective 9p24.1 amplification, increased PD-1 ligand expression, and further induction via JAK2 in nodular sclerosing Hodgkin lymphoma and primary mediastinal large B-cell lymphoma. Blood.

[28] Steidl C., Shah S.P., Woolcock B.W., Rui L., Kawahara M., Farinha P., Johnson N.A., Zhao Y., Telenius A., Neriah S.B. (2011). MHC class II transactivator CIITA is a recurrent gene fusion partner in lymphoid cancers. Nature.

[29] Hao Y., Chapuy B., Monti S., Sun H.H., Rodig S.J., Shipp M.A. (2014). Selective JAK2 inhibition specifically decreases Hodgkin lymphoma and mediastinal large B-cell lymphoma growth in vitro and in vivo. Clin. Cancer Res..

[30] Diaz T., Navarro A., Ferrer G., Gel B., Gaya A., Artells R., Bellosillo B., Garcia-Garcia M., Serrano S., Martinez A. (2011). Lestaurtinib inhibition of the Jak/STAT signaling pathway in hodgkin lymphoma inhibits proliferation and induces apoptosis. PLoS ONE.

[31] Holtick U., Vockerodt M., Pinkert D., Schoof N., Sturzenhofecker B., Kussebi N., Lauber K., Wesselborg S., Loffler D., Horn F. (2005). STAT3 is essential for Hodgkin lymphoma cell proliferation and is a target of tyrphostin AG17 which confers sensitization for apoptosis. Leukemia.

[32] Tiacci E., Ladewig E., Schiavoni G., Penson A., Fortini E., Pettirossi V., Wang Y., Rosseto A., Venanzi A., Vlasevska S. (2018). Pervasive mutations of JAK-STAT pathway genes in classical Hodgkin lymphoma. Blood.

[33] Piris M.A., Medeiros L.J., Chang K.C. (2020). Hodgkin lymphoma: A review of pathological features and recent advances in pathogenesis. Pathology.

[34] Panjwani P.K., Charu V., DeLisser M., Molina-Kirsch H., Natkunam Y., Zhao S. (2018). Programmed death-1 ligands PD-L1 and PD-L2 show distinctive and restricted patterns of expression in lymphoma subtypes. Hum. Pathol..

[35] Green M.R., Rodig S., Juszczynski P., Ouyang J., Sinha P., O'Donnell E., Neuberg D., Shipp M.A. (2012). Constitutive AP-1 activity and EBV infection induce PD-L1 in Hodgkin lymphomas and posttransplant lymphoproliferative disorders: Implications for targeted therapy. Clin. Cancer Res..

[36] Steidl C., Lee T., Shah S.P., Farinha P., Han G., Nayar T., Delaney A., Jones S.J., Iqbal J., Weisenburger D.D. (2010). Tumor-associated macrophages and survival in classic Hodgkin's lymphoma. N. Engl. J. Med..

[37] Tan K.L., Scott D.W., Hong F., Kahl B.S., Fisher R.I., Bartlett N.L., Advani R.H., Buckstein R., Rimsza L.M., Connors J.M. (2012). Tumor-associated macrophages predict inferior outcomes in classic Hodgkin lymphoma: A correlative study from the E2496 Intergroup trial. Blood.

[38] Vari F., Arpon D., Keane C., Hertzberg M.S., Talaulikar D., Jain S., Cui Q., Han E., Tobin J., Bird R. (2018). Immune evasion via PD-1/PD-L1 on NK cells and monocyte/macrophages is more prominent in Hodgkin lymphoma than DLBCL. Blood.

[39] Kawashima M., Carreras J., Higuchi H., Kotaki R., Hoshina T., Okuyama K., Suzuki N., Kakizaki M., Miyatake Y., Ando K. (2020). PD-L1/L2 protein levels rapidly increase on monocytes via trogocytosis from tumor cells in classical Hodgkin lymphoma. Leukemia.

[40] Carey C.D., Gusenleitner D., Lipschitz M., Roemer M.G.M., Stack E.C., Gjini E., Hu X., Redd R., Freeman G.J., Neuberg D. (2017). Topological analysis reveals a PD-L1-associated microenvironmental niche for Reed-Sternberg cells in Hodgkin lymphoma. Blood.

[41] Gordon L.I., Hong F., Fisher R.I., Bartlett N.L., Connors J.M., Gascoyne R.D., Wagner H., Stiff P.J., Cheson B.D., Gospodarowicz M. (2013). Randomized phase III trial of ABVD versus Stanford V with or without radiation therapy in locally extensive and advanced-stage Hodgkin lymphoma: An intergroup study coordinated by the Eastern Cooperative Oncology Group (E2496). J. Clin. Oncol..

[42] Borchmann P., Haverkamp H., Diehl V., Cerny T., Markova J., Ho A.D., Eich H.T., Mueller-Hermelink H.K., Kanz L., Greil R. (2011). Eight cycles of escalated-dose BEACOPP compared with four cycles of escalated-dose BEACOPP followed by four cycles of baseline-dose BEACOPP with or without radiotherapy in patients with advanced-stage hodgkin's lymphoma: Final analysis of the HD12 trial of the German Hodgkin Study Group. J. Clin. Oncol..

[43] Shanbhag S., Ambinder R.F. (2018). Hodgkin lymphoma: A review and update on recent progress. CA Cancer J. Clin..

[44] Gerrie A.S., Power M.M., Shepherd J.D., Savage K.J., Sehn L.H., Connors J.M. (2014). Chemoresistance can be overcome with high-dose chemotherapy and autologous stem-cell transplantation for relapsed and refractory Hodgkin lymphoma. Ann. Oncol..

[45] Ansell S.M., Lesokhin A.M., Borrello I., Halwani A., Scott E.C., Gutierrez M., Schuster S.J., Millenson M.M., Cattry D., Freeman G.J. (2015). PD-1 blockade with nivolumab in relapsed or refractory Hodgkin’s lymphoma. N. Engl. J. Med..

[46] Chen R., Zinzani P.L., Fanale M.A., Armand P., Johnson N.A., Brice P., Radford J., Ribrag V., Molin D., Vassilakopoulos T.P. (2017). Phase II Study of the Efficacy and Safety of Pembrolizumab for Relapsed/Refractory Classic Hodgkin Lymphoma. J. Clin. Oncol..

[47] Chen R., Zinzani P.L., Lee H.J., Armand P., Johnson N.A., Brice P., Radford J., Ribrag V., Molin D., Vassilakopoulos T.P. (2019). Pembrolizumab in relapsed or refractory Hodgkin lymphoma: 2-year follow-up of KEYNOTE-087. Blood.

[48] Kuruvilla J., Ramchandren R., Santoro A., Paszkiewicz-Kozik E., Gasiorowski R., Johnson N.A., Fogliatto L.M., Goncalves I., de Oliveira J.S.R., Buccheri V. (2021). Pembrolizumab versus brentuximab vedotin in relapsed or refractory classical Hodgkin lymphoma (KEYNOTE-204): An interim analysis of a multicentre, randomised, open-label, phase 3 study. Lancet Oncol..

[49] Shi Y., Su H., Song Y., Jiang W., Sun X., Qian W., Zhang W., Gao Y., Jin Z., Zhou J. (2019). Safety and activity of sintilimab in patients with relapsed or refractory classical Hodgkin lymphoma (ORIENT-1): A multicentre, single-arm, phase 2 trial. Lancet Haematol..

[50] Song Y., Gao Q., Zhang H., Fan L., Zhou J., Zou D., Li W., Yang H., Liu T., Wang Q. (2020). Treatment of relapsed or refractory classical Hodgkin lymphoma with the anti-PD-1, tislelizumab: Results of a phase 2, single-arm, multicenter study. Leukemia.

[51] Song Y., Wu J., Chen X., Lin T., Cao J., Liu Y., Zhao Y., Jin J., Huang H., Hu J. (2019). A Single-Arm, Multicenter, Phase II Study of Camrelizumab in Relapsed or Refractory Classical Hodgkin Lymphoma. Clin. Cancer Res..

[52] Herrera A.F., Burton C., Radford J., Miall F., Townsend W., Santoro A., Zinzani P.L., Lewis D., Fowst C., Brar S. (2021). Avelumab in relapsed/refractory classical Hodgkin lymphoma: Phase 1b results from the JAVELIN Hodgkins trial. Blood Adv..

[53] Allen P.B., Savas H., Evens A.M., Advani R.H., Palmer B., Pro B., Karmali R., Mou E., Bearden J., Dillehay G. (2021). Pembrolizumab followed by AVD in untreated early unfavorable and advanced-stage classical Hodgkin lymphoma. Blood.

[54] Herrera A.F., Moskowitz A.J., Bartlett N.L., Vose J.M., Ramchandren R., Feldman T.A., LaCasce A.S., Ansell S.M., Moskowitz C.H., Fenton K. (2018). Interim results of brentuximab vedotin in combination with nivolumab in patients with relapsed or refractory Hodgkin lymphoma. Blood.

[55] Alizadeh A.A., Eisen M.B., Davis R.E., Ma C., Lossos I.S., Rosenwald A., Boldrick J.C., Sabet H., Tran T., Yu X. (2000). Distinct types of diffuse large B-cell lymphoma identified by gene expression profiling. Nature.

[56] Wright G.W., Huang D.W., Phelan J.D., Coulibaly Z.A., Roulland S., Young R.M., Wang J.Q., Schmitz R., Morin R.D., Tang J. (2020). A Probabilistic Classification Tool for Genetic Subtypes of Diffuse Large B Cell Lymphoma with Therapeutic Implications. Cancer Cell.

[57] Lacy S.E., Barrans S.L., Beer P.A., Painter D., Smith A.G., Roman E., Cooke S.L., Ruiz C., Glover P., Van Hoppe S.J.L. (2020). Targeted sequencing in DLBCL, molecular subtypes, and outcomes: A Haematological Malignancy Research Network report. Blood.

[58] Kwon D., Kim S., Kim P.J., Go H., Nam S.J., Paik J.H., Kim Y.A., Kim T.M., Heo D.S., Kim C.W. (2016). Clinicopathological analysis of programmed cell death 1 and programmed cell death ligand 1 expression in the tumour microenvironments of diffuse large B cell lymphomas. Histopathology.

[59] Andorsky D.J., Yamada R.E., Said J., Pinkus G.S., Betting D.J., Timmerman J.M. (2011). Programmed death ligand 1 is expressed by non-hodgkin lymphomas and inhibits the activity of tumor-associated T cells. Clin. Cancer Res..

[60] Georgiou K., Chen L., Berglund M., Ren W., de Miranda N.F., Lisboa S., Fangazio M., Zhu S., Hou Y., Wu K. (2016). Genetic basis of PD-L1 overexpression in diffuse large B-cell lymphomas. Blood.

[61] Godfrey J., Tumuluru S., Bao R., Leukam M., Venkataraman G., Phillip J., Fitzpatrick C., McElherne J., MacNabb B.W., Orlowski R. (2019). PD-L1 gene alterations identify a subset of diffuse large B-cell lymphoma harboring a T-cell-inflamed phenotype. Blood.

[62] Kataoka K., Shiraishi Y., Takeda Y., Sakata S., Matsumoto M., Nagano S., Maeda T., Nagata Y., Kitanaka A., Mizuno S. (2016). Aberrant PD-L1 expression through 3'-UTR disruption in multiple cancers. Nature.

[63] Kwon H.J., Yang J.M., Lee J.O., Lee J.S., Paik J.H. (2018). Clinicopathologic implication of PD-L1 and phosphorylated STAT3 expression in diffuse large B cell lymphoma. J. Transl. Med..

[64] Tamma R., Ingravallo G., Albano F., Gaudio F., Annese T., Ruggieri S., Lorusso L., Errede M., Maiorano E., Specchia G. (2019). STAT-3 RNAscope Determination in Human Diffuse Large B-Cell Lymphoma. Transl. Oncol..

[65] Ok C.Y., Chen J., Xu-Monette Z.Y., Tzankov A., Manyam G.C., Li L., Visco C., Montes-Moreno S., Dybkaer K., Chiu A. (2014). Clinical implications of phosphorylated STAT3 expression in De Novo diffuse large B-cell lymphoma. Clin. Cancer Res..

[66] Gupta M., Han J.J., Stenson M., Maurer M., Wellik L., Hu G., Ziesmer S., Dogan A., Witzig T.E. (2012). Elevated serum IL-10 levels in diffuse large B-cell lymphoma: A mechanism of aberrant JAK2 activation. Blood.

[67] Coiffier B., Lepage E., Briere J., Herbrecht R., Tilly H., Bouabdallah R., Morel P., Van Den Neste E., Salles G., Gaulard P. (2002). CHOP chemotherapy plus rituximab compared with CHOP alone in elderly patients with diffuse large-B-cell lymphoma. N. Engl. J. Med..

[68] Pfreundschuh M., Trumper L., Osterborg A., Pettengell R., Trneny M., Imrie K., Ma D., Gill D., Walewski J., Zinzani P.L. (2006). CHOP-like chemotherapy plus rituximab versus CHOP-like chemotherapy alone in young patients with good-prognosis diffuse large-B-cell lymphoma: A randomised controlled trial by the MabThera International Trial (MInT) Group. Lancet Oncol..

[69] Ansell S.M., Minnema M.C., Johnson P., Timmerman J.M., Armand P., Shipp M.A., Rodig S.J., Ligon A.H., Roemer M.G.M., Reddy N. (2019). Nivolumab for Relapsed/Refractory Diffuse Large B-Cell Lymphoma in Patients Ineligible for or Having Failed Autologous Transplantation: A Single-Arm, Phase II Study. J. Clin. Oncol..

[70] Frigault M.J., Armand P., Redd R.A., Jeter E., Merryman R.W., Coleman K.C., Herrera A.F., Dahi P., Nieto Y., LaCasce A.S. (2020). PD-1 blockade for diffuse large B-cell lymphoma after autologous stem cell transplantation. Blood Adv..

[71] Chong E.A., Melenhorst J.J., Lacey S.F., Ambrose D.E., Gonzalez V., Levine B.L., June C.H., Schuster S.J. (2017). PD-1 blockade modulates chimeric antigen receptor (CAR)-modified T cells: Refueling the CAR. Blood.

[72] Mottok A., Hung S.S., Chavez E.A., Woolcock B., Telenius A., Chong L.C., Meissner B., Nakamura H., Rushton C., Vigano E. (2019). Integrative genomic analysis identifies key pathogenic mechanisms in primary mediastinal large B-cell lymphoma. Blood.

[73] Rosenwald A., Wright G., Leroy K., Yu X., Gaulard P., Gascoyne R.D., Chan W.C., Zhao T., Haioun C., Greiner T.C. (2003). Molecular diagnosis of primary mediastinal B cell lymphoma identifies a clinically favorable subgroup of diffuse large B cell lymphoma related to Hodgkin lymphoma. J. Exp. Med..

[74] Savage K.J., Monti S., Kutok J.L., Cattoretti G., Neuberg D., De Leval L., Kurtin P., Dal Cin P., Ladd C., Feuerhake F. (2003). The molecular signature of mediastinal large B-cell lymphoma differs from that of other diffuse large B-cell lymphomas and shares features with classical Hodgkin lymphoma. Blood.

[75] Dubois S., Viailly P.J., Mareschal S., Bohers E., Bertrand P., Ruminy P., Maingonnat C., Jais J.P., Peyrouze P., Figeac M. (2016). Next-Generation Sequencing in Diffuse Large B-Cell Lymphoma Highlights Molecular Divergence and Therapeutic Opportunities: A LYSA Study. Clin. Cancer Res..

[76] Shi M., Roemer M.G., Chapuy B., Liao X., Sun H., Pinkus G.S., Shipp M.A., Freeman G.J., Rodig S.J. (2014). Expression of programmed cell death 1 ligand 2 (PD-L2) is a distinguishing feature of primary mediastinal (thymic) large B-cell lymphoma and associated with PDCD1LG2 copy gain. Am. J. Surg. Pathol..

[77] Twa D.D., Chan F.C., Ben-Neriah S., Woolcock B.W., Mottok A., Tan K.L., Slack G.W., Gunawardana J., Lim R.S., McPherson A.W. (2014). Genomic rearrangements involving programmed death ligands are recurrent in primary mediastinal large B-cell lymphoma. Blood.

[78] Chong L.C., Twa D.D., Mottok A., Ben-Neriah S., Woolcock B.W., Zhao Y., Savage K.J., Marra M.A., Scott D.W., Gascoyne R.D. (2016). Comprehensive characterization of programmed death ligand structural rearrangements in B-cell non-Hodgkin lymphomas. Blood.

[79] Savage K.J. (2021). Primary mediastinal Large B-cell Lymphoma. Blood.

[80] Zinzani P.L., Ribrag V., Moskowitz C.H., Michot J.M., Kuruvilla J., Balakumaran A., Zhang Y., Chlosta S., Shipp M.A., Armand P. (2017). Safety and tolerability of pembrolizumab in patients with relapsed/refractory primary mediastinal large B-cell lymphoma. Blood.

[81] Armand P., Rodig S., Melnichenko V., Thieblemont C., Bouabdallah K., Tumyan G., Ozcan M., Portino S., Fogliatto L., Caballero M.D. (2019). Pembrolizumab in Relapsed or Refractory Primary Mediastinal Large B-Cell Lymphoma. J. Clin. Oncol..

[82] Fakhri B., Ai W. (2021). Current and emerging treatment options in primary mediastinal B-cell lymphoma. Ther. Adv. Hematol..

[83] Zinzani P.L., Santoro A., Gritti G., Brice P., Barr P.M., Kuruvilla J., Cunningham D., Kline J., Johnson N.A., Mehta-Shah N. (2019). Nivolumab Combined With Brentuximab Vedotin for Relapsed/Refractory Primary Mediastinal Large B-Cell Lymphoma: Efficacy and Safety From the Phase II CheckMate 436 Study. J. Clin. Oncol..

[84] Satou A., Nakamura S. (2021). EBV-positive B-cell lymphomas and lymphoproliferative disorders: Review from the perspective of immune escape and immunodeficiency. Cancer Med..

[85] Nicolae A., Pittaluga S., Abdullah S., Steinberg S.M., Pham T.A., Davies-Hill T., Xi L., Raffeld M., Jaffe E.S. (2015). EBV-positive large B-cell lymphomas in young patients: A nodal lymphoma with evidence for a tolerogenic immune environment. Blood.

[86] Yoon H., Park S., Ju H., Ha S.Y., Sohn I., Jo J., Do I.G., Min S., Kim S.J., Kim W.S. (2015). Integrated copy number and gene expression profiling analysis of Epstein-Barr virus-positive diffuse large B-cell lymphoma. Genes Chromosomes Cancer.

[87] Kataoka K., Miyoshi H., Sakata S., Dobashi A., Couronne L., Kogure Y., Sato Y., Nishida K., Gion Y., Shiraishi Y. (2019). Frequent structural variations involving programmed death ligands in Epstein-Barr virus-associated lymphomas. Leukemia.

[88] Marques-Piubelli M.L., Salas Y.I., Pachas C., Becker-Hecker R., Vega F., Miranda R.N. (2020). Epstein-Barr virus-associated B-cell lymphoproliferative disorders and lymphomas: A review. Pathology.

[89] Ok C.Y., Li L., Xu-Monette Z.Y., Visco C., Tzankov A., Manyam G.C., Montes-Moreno S., Dybkaer K., Chiu A., Orazi A. (2014). Prevalence and clinical implications of epstein-barr virus infection in de novo diffuse large B-cell lymphoma in Western countries. Clin. Cancer Res..

[90] Bourbon E., Maucort-Boulch D., Fontaine J., Mauduit C., Sesques P., Safar V., Ferrant E., Golfier C., Ghergus D., Karlin L. (2021). Clinicopathological features and survival in EBV-positive diffuse large B-cell lymphoma not otherwise specified. Blood Adv..

[91] Kim M., Lee J.O., Koh J., Kim T.M., Lee J.Y., Jeon Y.K., Keam B., Kim D.W., Lee J.S., Heo D.S. (2021). A phase II study of brentuximab vedotin in patients with relapsed or refractory Epstein-Barr virus-positive and CD30-positive lymphomas. Haematologica.

[92] Castillo J.J., Beltran B.E., Miranda R.N., Young K.H., Chavez J.C., Sotomayor E.M. (2018). EBV-positive diffuse large B-cell lymphoma, not otherwise specified: 2018 update on diagnosis, risk-stratification and management. Am. J. Hematol..

[93] Montes-Moreno S., Odqvist L., Diaz-Perez J.A., Lopez A.B., de Villambrosia S.G., Mazorra F., Castillo M.E., Lopez M., Pajares R., Garcia J.F. (2012). EBV-positive diffuse large B-cell lymphoma of the elderly is an aggressive post-germinal center B-cell neoplasm characterized by prominent nuclear factor-kB activation. Mod. Pathol..

[94] Kato H., Karube K., Yamamoto K., Takizawa J., Tsuzuki S., Yatabe Y., Kanda T., Katayama M., Ozawa Y., Ishitsuka K. (2014). Gene expression profiling of Epstein-Barr virus-positive diffuse large B-cell lymphoma of the elderly reveals alterations of characteristic oncogenetic pathways. Cancer Sci..

[95] Chapman J.R., Bouska A.C., Zhang W., Alderuccio J.P., Lossos I.S., Rimsza L.M., Maguire A., Yi S., Chan W.C., Vega F. (2021). EBV-positive HIV-associated diffuse large B cell lymphomas are characterized by JAK/STAT (STAT3) pathway mutations and unique clinicopathologic features. Br. J. Haematol..

[96] Jiang X.N., Yu B.H., Yan W.H., Lee J., Zhou X.Y., Li X.Q. (2020). Epstein-Barr virus-positive diffuse large B-cell lymphoma features disrupted antigen capture/presentation and hijacked T-cell suppression. Oncoimmunology.

[97] Quan L., Chen X., Liu A., Zhang Y., Guo X., Yan S., Liu Y. (2015). PD-1 Blockade Can Restore Functions of T-Cells in Epstein-Barr Virus-Positive Diffuse Large B-Cell Lymphoma In Vitro. PLoS ONE.

[98] Yu M., Zhang Q., Xu S., Yin T., Li F. (2022). Successful treatment of refractory retroperitoneal Epstein-Barr virus-positive diffuse large B-cell lymphoma with secondary hemophagocytic syndrome by sequential combination regimen of PD-1 blockade and chimeric antigen receptor T cells: A case report. Anticancer Drugs.

[99] Yilmaz E., Lakhotia R., Pittaluga S., Muppidi J.R., Phelan J.D., Evans S., Pradhan A., Hillsman A., Steinberg S.M., Jaffe E.S. (2021). Phase 2 Study of Nivolumab in Epstein-Barr Virus (EBV)-Positive Lymphoproliferative Disorders and EBV-Positive Non-Hodgkin Lymphomas. Blood.

[100] Morris S.W., Kirstein M.N., Valentine M.B., Dittmer K.G., Shapiro D.N., Saltman D.L., Look A.T. (1994). Fusion of a kinase gene, ALK, to a nucleolar protein gene, NPM, in non-Hodgkin's lymphoma. Science.

[101] Kaneko Y., Frizzera G., Edamura S., Maseki N., Sakurai M., Komada Y., Sakurai M., Tanaka H., Sasaki M., Suchi T. (1989). A novel translocation, t(2;5)(p23;q35), in childhood phagocytic large T-cell lymphoma mimicking malignant histiocytosis. Blood.

[102] Mason D.Y., Bastard C., Rimokh R., Dastugue N., Huret J.L., Kristoffersson U., Magaud J.P., Nezelof C., Tilly H., Vannier J.P. (1990). CD30-positive large cell lymphomas (‘Ki-1 lymphoma’) are associated with a chromosomal translocation involving 5q35. Br. J. Haematol..

[103] Crescenzo R., Abate F., Lasorsa E., Tabbo F., Gaudiano M., Chiesa N., Di Giacomo F., Spaccarotella E., Barbarossa L., Ercole E. (2015). Convergent mutations and kinase fusions lead to oncogenic STAT3 activation in anaplastic large cell lymphoma. Cancer Cell.

[104] Parrilla Castellar E.R., Jaffe E.S., Said J.W., Swerdlow S.H., Ketterling R.P., Knudson R.A., Sidhu J.S., Hsi E.D., Karikehalli S., Jiang L. (2014). ALK-negative anaplastic large cell lymphoma is a genetically heterogeneous disease with widely disparate clinical outcomes. Blood.

[105] Vasmatzis G., Johnson S.H., Knudson R.A., Ketterling R.P., Braggio E., Fonseca R., Viswanatha D.S., Law M.E., Kip N.S., Ozsan N. (2012). Genome-wide analysis reveals recurrent structural abnormalities of TP63 and other p53-related genes in peripheral T-cell lymphomas. Blood.

[106] Marzec M., Zhang Q., Goradia A., Raghunath P.N., Liu X., Paessler M., Wang H.Y., Wysocka M., Cheng M., Ruggeri B.A. (2008). Oncogenic kinase NPM/ALK induces through STAT3 expression of immunosuppressive protein CD274 (PD-L1, B7-H1). Proc. Natl. Acad. Sci. USA.

[107] Shen J., Li S., Medeiros L.J., Lin P., Wang S.A., Tang G., Yin C.C., You M.J., Khoury J.D., Iyer S.P. (2019). PD-L1 expression is associated with ALK positivity and STAT3 activation, but not outcome in patients with systemic anaplastic large cell lymphoma. Mod. Pathol..

[108] Manso R., Rodriguez-Perales S., Torres-Ruiz R., Santonja C., Rodriguez-Pinilla S.M. (2021). PD-L1 expression in peripheral T-cell lymphomas is not related to either PD-L1 gene amplification or rearrangements. Leuk Lymphoma.

[109] Neuwelt A., Al-Juhaishi T., Davila E., Haverkos B. (2020). Enhancing antitumor immunity through checkpoint blockade as a therapeutic strategy in T-cell lymphomas. Blood Adv..

[110] Onaindia A., de Villambrosia S.G., Prieto-Torres L., Rodriguez-Pinilla S.M., Montes-Moreno S., Gonzalez-Vela C., Piris M.A. (2019). DUSP22-rearranged anaplastic lymphomas are characterized by specific morphological features and a lack of cytotoxic and JAK/STAT surrogate markers. Haematologica.

[111] Hapgood G., Ben-Neriah S., Mottok A., Lee D.G., Robert K., Villa D., Sehn L.H., Connors J.M., Gascoyne R.D., Feldman A.L. (2019). Identification of high-risk DUSP22-rearranged ALK-negative anaplastic large cell lymphoma. Br. J. Haematol..

[112] Luchtel R.A., Dasari S., Oishi N., Pedersen M.B., Hu G., Rech K.L., Ketterling R.P., Sidhu J., Wang X., Katoh R. (2018). Molecular profiling reveals immunogenic cues in anaplastic large cell lymphomas with DUSP22 rearrangements. Blood.

[113] Boi M., Rinaldi A., Kwee I., Bonetti P., Todaro M., Tabbo F., Piva R., Rancoita P.M., Matolcsy A., Timar B. (2013). PRDM1/BLIMP1 is commonly inactivated in anaplastic large T-cell lymphoma. Blood.

[114] Atsaves V., Tsesmetzis N., Chioureas D., Kis L., Leventaki V., Drakos E., Panaretakis T., Grander D., Medeiros L.J., Young K.H. (2017). PD-L1 is commonly expressed and transcriptionally regulated by STAT3 and MYC in ALK-negative anaplastic large-cell lymphoma. Leukemia.

[115] Khoury J.D., Medeiros L.J., Rassidakis G.Z., Yared M.A., Tsioli P., Leventaki V., Schmitt-Graeff A., Herling M., Amin H.M., Lai R. (2003). Differential expression and clinical significance of tyrosine-phosphorylated STAT3 in ALK+ and ALK- anaplastic large cell lymphoma. Clin. Cancer Res..

[116] Andersson E.I., Bruck O., Braun T., Mannisto S., Saikko L., Lagstrom S., Ellonen P., Leppa S., Herling M., Kovanen P.E. (2020). STAT3 Mutation Is Associated with STAT3 Activation in CD30(+) ALK(-) ALCL. Cancers.

[117] Yamamoto R., Nishikori M., Tashima M., Sakai T., Ichinohe T., Takaori-Kondo A., Ohmori K., Uchiyama T. (2009). B7-H1 expression is regulated by MEK/ERK signaling pathway in anaplastic large cell lymphoma and Hodgkin lymphoma. Cancer Sci..

[118] Pearson J.D., Lee J.K., Bacani J.T., Lai R., Ingham R.J. (2012). NPM-ALK: The Prototypic Member of a Family of Oncogenic Fusion Tyrosine Kinases. J. Signal Transduct..

[119] Chan T.S., Khong P.L., Kwong Y.L. (2016). Pembrolizumab for relapsed anaplastic large cell lymphoma after allogeneic haematopoietic stem cell transplantation: Efficacy and safety. Ann. Hematol..

[120] Rigaud C., Abbou S., Minard-Colin V., Geoerger B., Scoazec J.Y., Vassal G., Jaff N., Heuberger L., Valteau-Couanet D., Brugieres L. (2018). Efficacy of nivolumab in a patient with systemic refractory ALK+ anaplastic large cell lymphoma. Pediatr. Blood Cancer.

[121] Hebart H., Lang P., Woessmann W. (2016). Nivolumab for Refractory Anaplastic Large Cell Lymphoma: A Case Report. Ann. Intern Med..

[122] Iyer S.P., Xu J., Becnel M.R., Nair R., Steiner R., Feng L., Lee H.J., Strati P., Ahmed S., Parmar S. (2020). A Phase II Study of Pembrolizumab in Combination with Romidepsin Demonstrates Durable Responses in Relapsed or Refractory T-Cell Lymphoma (TCL). Blood.

[123] Bednarska K., Nath K., Nicol W., Gandhi M.K. (2021). Immunity reloaded: Deconstruction of the PD-1 axis in B cell lymphomas. Blood Rev..

[124] Anand K., Ensor J., Pingali S.R., Hwu P., Duvic M., Chiang S., Miranda R., Zu Y., Iyer S. (2020). T-cell lymphoma secondary to checkpoint inhibitor therapy. J. Immunother. Cancer.

[125] Oishi N., Brody G.S., Ketterling R.P., Viswanatha D.S., He R., Dasari S., Mai M., Benson H.K., Sattler C.A., Boddicker R.L. (2018). Genetic subtyping of breast implant-associated anaplastic large cell lymphoma. Blood.

[126] Clemens M.W., Medeiros L.J., Butler C.E., Hunt K.K., Fanale M.A., Horwitz S., Weisenburger D.D., Liu J., Morgan E.A., Kanagal-Shamanna R. (2016). Complete Surgical Excision Is Essential for the Management of Patients With Breast Implant-Associated Anaplastic Large-Cell Lymphoma. J. Clin. Oncol..

[127] Evans M.G., Medeiros L.J., Marques-Piubelli M.L., Wang H.Y., Ortiz-Hidalgo C., Pina-Oviedo S., Morine A., Clemens M.W., Hunt K.K., Iyer S. (2021). Breast implant-associated anaplastic large cell lymphoma: Clinical follow-up and analysis of sequential pathologic specimens of untreated patients shows persistent or progressive disease. Mod. Pathol..

[128] Laurent C., Nicolae A., Laurent C., Le Bras F., Haioun C., Fataccioli V., Amara N., Adelaide J., Guille A., Schiano J.M. (2020). Gene alterations in epigenetic modifiers and JAK-STAT signaling are frequent in breast implant-associated ALCL. Blood.

[129] Blombery P., Thompson E., Ryland G.L., Joyce R., Byrne D.J., Khoo C., Lade S., Hertzberg M., Hapgood G., Marlton P. (2018). Frequent activating STAT3 mutations and novel recurrent genomic abnormalities detected in breast implant-associated anaplastic large cell lymphoma. Oncotarget.

[130] Quesada A.E., Zhang Y., Ptashkin R., Ho C., Horwitz S., Benayed R., Dogan A., Arcila M.E. (2021). Next generation sequencing of breast implant-associated anaplastic large cell lymphomas reveals a novel STAT3-JAK2 fusion among other activating genetic alterations within the JAK-STAT pathway. Breast J..

[131] Tabanelli V., Corsini C., Fiori S., Agostinelli C., Calleri A., Orecchioni S., Melle F., Motta G., Rotili A., Di Napoli A. (2019). Recurrent PDL1 expression and PDL1 (CD274) copy number alterations in breast implant-associated anaplastic large cell lymphomas. Hum. Pathol..

